# Comprehensive Transcriptome Assembly of Chickpea (*Cicer arietinum* L.) Using Sanger and Next Generation Sequencing Platforms: Development and Applications

**DOI:** 10.1371/journal.pone.0086039

**Published:** 2014-01-23

**Authors:** Himabindu Kudapa, Sarwar Azam, Andrew G. Sharpe, Bunyamin Taran, Rong Li, Benjamin Deonovic, Connor Cameron, Andrew D. Farmer, Steven B. Cannon, Rajeev K. Varshney

**Affiliations:** 1 Research Program on Grain Legumes, International Crops Research Institute for the Semi-Arid Tropics (ICRISAT), Patancheru, Andhra Pradesh, India; 2 National Research Council Canada (NRC-CNRC), Saskatoon, Saskatchewan, Canada; 3 Department of Plant Sciences, University of Saskatchewan, Saskatoon, Saskatchewan, Canada; 4 Department of Agronomy, University of Iowa, Ames, Iowa, United States of America; 5 National Center for Genome Resources (NCGR), Santa Fe, New Mexico, United States of America; 6 United States Department of Agriculture–Agricultural Research Service (USDA–ARS), Corn Insects and Crop Genetics Research Unit (USDA-ARS-CICGRU), Ames, Iowa, United States of America; 7 CGIAR Generation Challenge Programme (GCP), c/o CIMMYT, Mexico DF, Mexico; University of Guelph, Canada

## Abstract

A comprehensive transcriptome assembly of chickpea has been developed using 134.95 million Illumina single-end reads, 7.12 million single-end FLX/454 reads and 139,214 Sanger expressed sequence tags (ESTs) from >17 genotypes. This hybrid transcriptome assembly, referred to as *Cicer arietinum* Transcriptome Assembly version 2 (CaTA v2, available at http://data.comparative-legumes.org/transcriptomes/cicar/lista_cicar-201201), comprising 46,369 transcript assembly contigs (TACs) has an N50 length of 1,726 bp and a maximum contig size of 15,644 bp. Putative functions were determined for 32,869 (70.8%) of the TACs and gene ontology assignments were determined for 21,471 (46.3%). The new transcriptome assembly was compared with the previously available chickpea transcriptome assemblies as well as to the chickpea genome. Comparative analysis of CaTA v2 against transcriptomes of three legumes - *Medicago*, soybean and common bean, resulted in 27,771 TACs common to all three legumes indicating strong conservation of genes across legumes. CaTA v2 was also used for identification of simple sequence repeats (SSRs) and intron spanning regions (ISRs) for developing molecular markers. ISRs were identified by aligning TACs to the *Medicago* genome, and their putative mapping positions at chromosomal level were identified using transcript map of chickpea. Primer pairs were designed for 4,990 ISRs, each representing a single contig for which predicted positions are inferred and distributed across eight linkage groups. A subset of randomly selected ISRs representing all eight chickpea linkage groups were validated on five chickpea genotypes and showed 20% polymorphism with average polymorphic information content (PIC) of 0.27. In summary, the hybrid transcriptome assembly developed and novel markers identified can be used for a variety of applications such as gene discovery, marker-trait association, diversity analysis etc., to advance genetics research and breeding applications in chickpea and other related legumes.

## Introduction

Chickpea (*Cicer arietinum*), is an important legume crop in the semi-arid tropics and ranks third in total yield among seed legume crops globally, after soybean and common bean (www.fao.org). It is a rich source of protein (20–25%) and enhances soil fertility by biological nitrogen fixation [1]–[3]. It is an important system for legume genetics and genomics research, with a small (740 Mb) and diploid (2n = 2x = 16) genome [4]. Annual world production of chickpea is about 9.8 million tons, and India alone contributes 68.6% of the world production (FAOSTAT, 2009; http://faostat.fao.org). Chickpea yields are limited by several abiotic stresses (e.g. drought, salinity, heat) and biotic stresses (e.g. Ascochyta blight, *Fusarium* wilt and pod borer). Addressing these stresses is critical to enhance crop productivity of chickpea. While efforts have been underway through conventional methods [5]–[6], molecular approaches, through genomics-assisted breeding [7] also have a great potential when coupled with conventional breeding.

Development of genomic resources remains a vital component for molecular or genomics-assisted breeding. Unfortunately, development of genomic resources for this economically important food legume crop has remained slow until recently and as a result genomics-assisted breeding has not been used effectively in the crop. In recent years, however, next generation sequencing (NGS) efforts and their use in genomics research, have greatly improved chickpea genomic resources [8]–[14]. Though several types of molecular markers and a number of genetic linkage maps have been available for some time, other genomic resources such as transcriptomic and genomic sequences and large SSR and SNP collections have become available only recently. These include 20,162 drought and salinity ESTs [15], 34,760 transcripts [12], 3,062 unigenes [16], a first-generation transcript map based on 126 loci [17], and a larger genetic map comprised of 1,291 loci [11]. Additionally, 435,018 FLX/454 reads and ∼37 million Illumina tags have been generated [10]. The 435,018 FLX/454 reads along with 21,491 Sanger ESTs available at that time were merged to generate the first version of chickpea transcriptome assembly (CaTA v1) comprised of 103,215 TUSs (Tentative Unique Sequences) [10]. A new transcript map comprising 1,328 loci has also been generated [9]. Furthermore at the time of writing this MS, draft genome sequence has become available for two types of chickpea: *kabuli* [18] and *desi* [19].

In this study, we used several chickpea Sanger EST collections, together with sequence from two different NGS platforms (Illumina and FLX/454), to produce a more extensive chickpea transcriptome assembly (CaTA v2). These ESTs/sequences were generated based on transcriptomic studies from >22 tissues (including diverse developmental stages and 8 stress responsive tissues) and >17 different chickpea genotypes [10], [12], [15], [20]. This assembly was analyzed for functional annotation (BLASTX comparisons against the UniProt database, UniRef90), Gene Ontology (GO) (UniProt database, UniProt-GO) descriptions, and molecular markers. The transcriptome assembly was also compared to the previously defined transcriptome assemblies and recently available chickpea genome sequences. Furthermore, it was compared to transcriptomes of other legumes (*Medicago*, soybean and common bean). Most importantly, CaTA v2 TACs (Transcript assembly Contigs) were aligned to the genome sequence of the model legume *Medicago truncatula* [21], a closely related sequenced legume species to chickpea. Anchoring points between chickpea and *Medicago* enabled identification of Intron Spanning Region (ISR) markers. Some ISR markers were also used for experimental validation.

## Results

### Defining a Comprehensive Transcriptome Assembly

Three datasets *viz.*, 134.95 million Illumina reads from one genotype (ICC 4958; Dataset I) [20], 7.12 million FLX/454 reads from nine genotypes (Datasets II) [10], [12], and 139,214 Sanger ESTs from >17 genotypes (Dataset III) ([15], [16], http://www.ncbi.nlm.nih.gov/dbEST/) (Table 1) were processed and assembled with ABySS [22], Newbler (http://www.454.com/products/analysis-software/) and MIRA [23] assemblers. For instance, initially Illumina reads (Dataset I) were assembled together using ABySS and FLX/454 reads (Dataset II) were assembled using Newbler. Subsequently, the pooled Illumina and FLX/454 assemblies were merged with Sanger ESTs using the MIRA assembler. Finally, the improved transcriptome assembly comprising of 48,668 TACs has been developed..

**Table 1:** Details on NGS (FLX/454 and Illumina) and Sanger sequencing datasets used for developing comprehensive chickpea transcriptome assembly (CaTA v2).

Dataset/sequencingplatform	Genotype	Tissues	Source*	Number of reads
*Dataset I*				
Illumina GAII	ICC 4958	root+shoot+leaves+buds	NIPGR	65,900,072 [20]
Illumina GAII	ICC 4958	root+shoot	NIPGR	69,054,282 [20]
*Dataset II*				
Roche/454	ICC 4958	RNA from 5 different tissues	NIPGR	∼2,500,000 [12]
Roche/454	ICC 4958	22 different developmental stages	ICRISAT/JCVI	∼400,000 [10]
Roche/454	Amit	RNA from 5 different tissues^1^	NRC	496,109
Roche/454	CDC Frontier	-do-	NRC	490,245
Roche/454	CDC Xena	-do-	NRC	531,970
Roche/454	Cr5-10	-do-	NRC	610,889
Roche/454	ICC12512-1	-do-	NRC	507,801
Roche/454	ICCV96029	-do-	NRC	520,733
Roche/454	ILWC 118	-do-	NRC	560,321
Roche/454	Y9563-28	-do-	NRC	509,682
*Dataset III*				
Sanger Sequencing	AAFC	mix of tissues	NRC	30,537
Sanger Sequencing	CDC Frontier	mix of tissues	NRC	66,720
Sanger Sequencing	C235, Castellana,Digvijay, ICC 4958, ICC1882, ICC 3996, ICCV 2,JG 315, JG 11, JG 62,Pedrosillano, Pusa,Pusa 362, WR 315,XJ 209, Azad	mix of tissues	NCBI	41,984 ([15], [16], NCBI)

1 Tissues collected: a) 2-week old leaf, b) stem before flowering, c) 1-week-old etiolated seedling, d) mixed flower stages and e) developing seed at mixed stages.

* NIPGR- National Institute of Plant Genome Research, India; ICRISAT- International Crops Research Institute for the Semi-Arid Tropics, India; JCVI- J. Craig Venter Institute, USA; NRC- National Research Council Canada.

To check for microbial contamination and rRNA contamination in the developed assembly, all 48,668 TACs were analyzed by BLAST similarity searches against databases of bacterial genomes (ftp://ftp.ncbi.nlm.nih.gov/genomes/Bacteria/) and rRNAs collected from plant species available at NCBI. Only 471 TACs had significant hits to bacterial genomes and 1,828 showed hits to rRNA. These TACs were discarded from further analysis, leaving 46,369 TACs in the assembly. This assembly was designated as CaTA v2 for *Cicer arietinum* Transcriptome Assembly version 2 (http://data.comparative-legumes.org/transcriptomes/cicar/lista_cicar-201201). The CaTA v2 developed in this study has several improved characteristics as compared to available transcriptome assemblies [10], [12], [20]. For instance, the N50 of TACs in CaTA v2 is 1,726 bp as compared to 515 bp [10], 730 bp [20] and 1,671 bp [12] of available assemblies (Table 2). The largest TAC in CaTA v2 is 15,644 bp, which is almost five times larger than that in the CaTA v1, with 3,346 bp. Therefore the hybrid transcriptome assembly is of higher quality than available assemblies.

**Table 2:** Comparative analysis of chickpea transcriptome assemblies.

	Comprehensive TA (CaTA v2)	Agarwal et al. [25]	CaTA v1 Hiremathet al. [10]	Garg et al. [12]	Garg et al. [20]	Deokar et al. [16]	Varshney et al. [15]
Sequence data used	∼7 million 454 reads+∼100 million Illumina+∼150K Sanger ESTs	1.8 million 454 reads +121million Illumina reads	435,018 454 reads+∼37million Illumina reads+21,491 Sanger ESTs	∼2 million 454 reads+∼ 107 million Illuminareads	∼ 107 millionIllumina reads	5,494 Sanger ESTs	20,162 Sanger ESTs
Number of genotypesproviding sequence data	10 (where more than half of datawas from ICC 4958)	1 (ICCV 2)	4 (ICC 4958, ICC 1882,JG 11, ICCV 2)	1 (ICC 4958)	1 (ICC 4958)	2 (ICC 4958, ICC 1882) +20 RILs (10 Resistant and 10 Sensitive)	4 (ICC 4958, ICC 1882, JG 11, ICCV 2)
Programme(s) used forassembly	MIRA (Newbler+Abyss)	TGICL, Newbler, CLCGenomics Workbench	CAP3	TGICL(Newbler+Velvet)	Velvet	CAP3	CAP3
Total number of TACs	46,369	43,389 contigs	103,215 (44,845 contigsand 58,370 singletons)	34,760 contigs	74,651 contigs	3,062 (638 contigsand 2,424 singletons)	6,404 (1,590 contigs and 4,814 singletons)
N50 (bp)	1,726	1,653	364 (515 bp for contigs)	1,671	730	–	–
Largest contig (bp)	15,644	15,605	3,346	13,803	7,827	–	–
Shortest contig (bp)	100	100	51	100	100	–	–

We checked the completeness of CaTA v2 transcriptome assembly with the core eukaryotic gene-mapping approach (CEGMA) pipeline [24]. CEGMA analysis undertakes similarity search of the assembly with a set of 458 highly conserved eukaryotic ubiquitous genes from the euKaryotic Orthologous Groups (KOG) database that are supposed to be present in all eukaryotes. A total of 452 (>98%) KOGs transcripts were present in CaTA v2 either completely or partially, which provides an indication of the completeness or comprehensiveness of the transcriptome assembly. Furthermore, full length transcripts present in the CaTA v2 were assessed using the annotated gene set (28,269) of the reported *kabuli* genome. On comparison, 11,088 TACs of CaTA v2 covers complete (100%) coding DNA sequences (CDSs) and can be considered as full length transcripts.

The CaTA v2 was compared to the previously available transcriptome assemblies developed by Hiremath et al. (CaTA v1, [10]), Deokar et al. [16], Garg et al. [12], [20] and Agarwal et al. [25] (Table 2). In this context, TACs from the CaTA v2 were clustered together with tentative unique sequences (TUSs) from CaTA v1 and contigs from other mentioned transcriptome assemblies. On clustering at 90% sequence identity, a non-redundant set of 84,754 transcripts (or unigenes) including 32,162 clusters and 52,592 singletons were defined. A total of 38,131 TACs of the CaTA v2 were found present in 25,580 (79.5%) clusters. Interestingly, 10,428 (47.7%) such clusters have representative sequence (longest sequence) from CaTA v2. Similarly, out of 52,592 singletons, 8,238 and 28,702 singletons are from the CaTA v2 and CaTA v1, respectively. The remaining singletons are from the transcriptome assemblies of Agarwal et al. (13,862 singletons, [25]) and Garg et al. (17,90 singletons, [12], [20]). These analyses in brief indicate high quality of CaTA v2 as even after combining five assemblies, 47.7% of cluster representative sequence has come from CaTA v2.

### Functional Annotation and Categorization of Gene Ontology (GO) Descriptions

**Figure 1:**
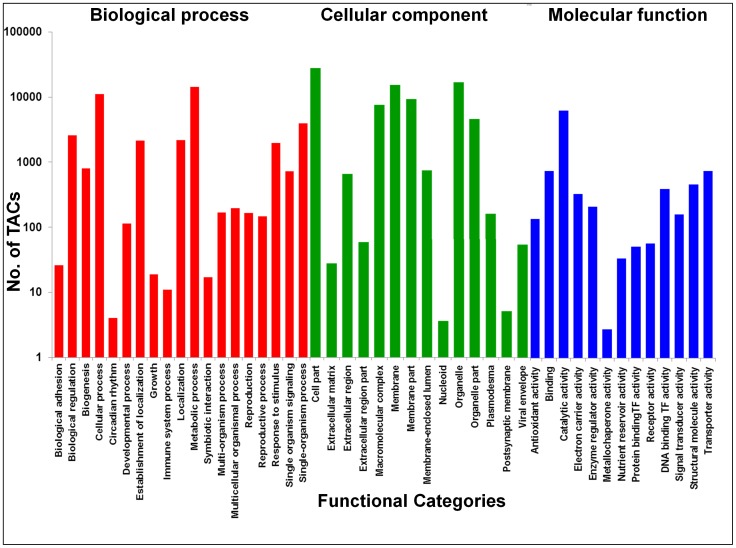
Functional categorization of chickpea Transcript Assembly Contigs (TACs) of the CaTA v2. Chickpea TACs representing the distribution of genes based on their annotations to terms in the GO were categorized hierarchically according to three principal gene ontologies, viz. biological processes, molecular functions and cellular components. The number of TACs representing each subcategory is shown in Y-axis.

Functional annotation of 46,369 TACs against the sequences of the UniProt database [26] showed that 32,869 (70.8%) of TACs had significant similarity at a threshold of E-value 1e-06. These were functionally categorized based on GO descriptions (UniProt database, UniProt-GO) and 21,471 TACs (46.3%) could be assigned to at least one of the GO terms. These were further assigned to three principal categories: molecular function (18,748), followed by biological function (17,924) and cellular component (8,671). The highest number of TACs fell into metabolic process (14,233 TACs), followed by catalytic activity (11,662 TACs), cellular process (11,049 TACs), cell part (5,963 TACs), single organism process (3961 TACs), organelle (3,914 TACs) and membrane (3,615 TACs) subcategories (Figure 1).

**Figure 2:**
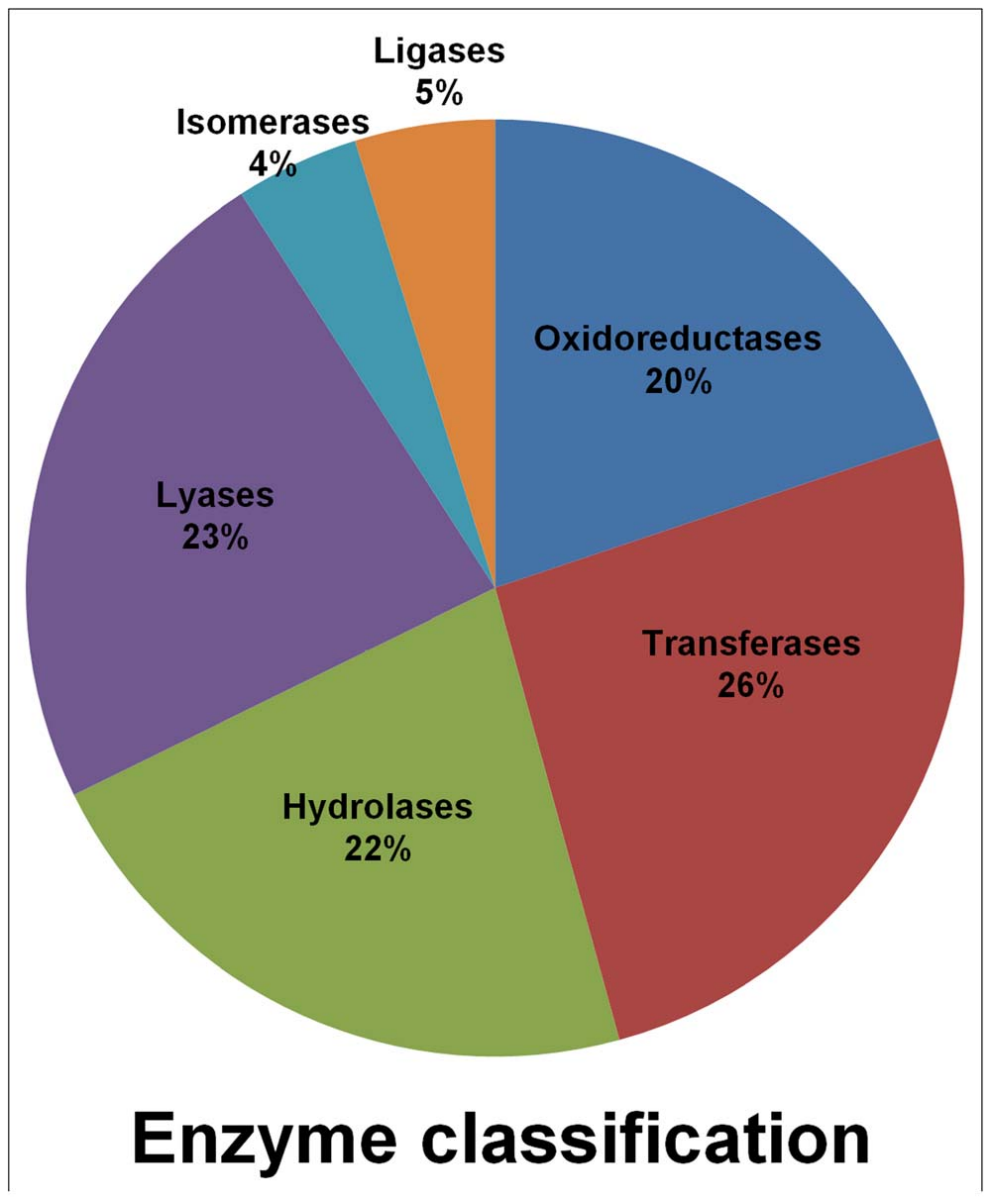
Enzyme classification of chickpea Transcript Assembly Contigs (TACs) among the six enzyme classes. The graph displays the proportion of genes belonging to each enzyme class.

Gene ontology classifications were also used to identify abiotic and biotic stress responsive genes. A large number of TACs (1,969) were found under the ‘response to stimulus’ subcategory which could be used to understand biological processes of different phenotypes under stress conditions. Additionally, Enzyme Commission (EC) IDs were retrieved for 1,615 TACs which would enable to map them to specific metabolic pathways. Maximum number of TACs belong to the enzyme class ‘transferases’ (419), followed by ‘lyases’ (375) and ‘hydrolases’ (356) (Figure 2). Leucine-rich repeat (LRR) receptor protein kinase EXS (UniProt ID B9SM68), Brassinosteroid insensitive 1-associated receptor kinase 1 (UniProt ID B9T8C3) and Brassinosteroid LRR receptor kinase (UniProt ID B9RLU0), have been assigned putatively to the oxido-reductase and transferase enzyme classes, while Brassinosteroid LRR receptor kinase (UniProt ID B9RLU0) has been assigned putatively to enzyme classes, transferase and hydrolase.

**Figure 3:**
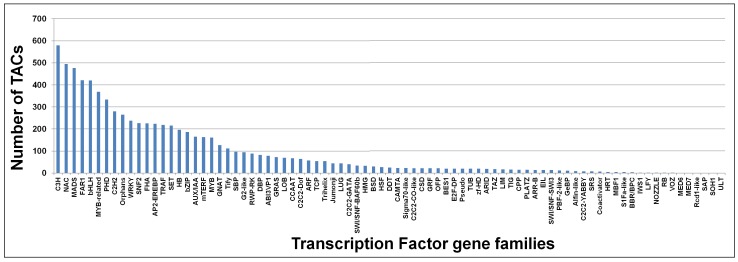
Distribution of chickpea transcripts in different transcription factor (TF) families. Based on conserved domain annotation, Transcript Assembly Contigs (TACs) showing significant annotation to transcription factors were classified.

In addition to above, transcription factors (TFs) which are involved in regulation of gene expression were identified for 7,722 TACs (16.65%) based on conserved domains. These putatively-assigned transcripts are distributed in 83 TF gene families. The C3H transcription factor gene family has highest number of transcripts (579), followed by NAC (495) and MADS (477). A few families like MED6, MED7, SAP, Rcd1-like, SOH1, and ULT have just one transcript (Figure 3).

### Comparison of CaTA v2 with the Chickpea Genome and Transcriptomes of Related Legumes

Recently draft genome sequence assemblies have become available for both *kabuli* [18] and *desi* [19] types. The CaTA v2 was compared against the above mentioned genome assemblies as well as annotated gene set of both the assemblies. On comparison with genome assemblies, 98% (45,391) and 84% (38,834) TACs were mapped onto *kabuli* genome with >50% and >90% coverage, respectively. On the other hand 90% (41,524) and 76% (35,270) TACs were mapped on to the *desi* genome at >50% and >90% coverage, respectively (Table S1). On comparison with annotated gene sets, 25,762 genes (out of 28,269; 91%) in *kabuli* genome and 19,033 (out of 27,571; 69%) in *desi* genome showed homology with CaTA v2 TACs. This indicates very less coverage of annotated genes of the CaTA v2 in the *desi* genome. To understand the reason for low coverage in *desi* genome as compared to *kabuli* genome, the set of 84,754 unigenes (clusters and singletons) identified after cluster analysis of five assemblies as mentioned earlier was compared with both the gene sets of *kabuli* and *desi* genomes. This comparison enhanced the coverage of gene set to 98% in *kabuli* genome but only to 72.4% in *desi* genome. In the other words, 610 (2%) genes remained uncovered in *kabuli* genome and 7,614 (27.6%) genes in *desi* genome. While analyzing uncovered genes (for a stretch of minimum 9 genes) in *desi* genome, several continuous segments were observed on both pseudomolecules as well as scaffolds that have clusters of uncovered genes. For instance, 15 continuous segments, not covered by genes were observed across all pseudomolecules except CaLG 07. The largest cluster of 28 uncovered genes (continuous) was found on CaLG 04. In context of scaffolds, a large number of clusters containing uncovered genes (7,094) including many clusters with large number of uncovered genes in long stretches were identified. These observations were verified further when uncovered genes from the gene set of the *desi* genome did not get hit even with 24.73 million Illumina transcript reads (after filtering from 31.02 million reads from SRR063784).

In addition, the developed transcriptome was compared with annotated transcriptomes of some legume species *viz*., *Medicago*, soybean and common bean (www.phytozome.net) Maximum (34,451) TACs showed hit to transcriptomes of soybean followed by common bean (33,105) and *Medicago* (30,811) at E-value 1e-10. Besides, 27,771 TACs were found common to all three legumes. At the stringent criteria of 80% query coverage and 80% identity, 12,298 TACs of CaTA v2 were aligned to soybean followed by 11,210 to *Medicago* and 9,605 to common bean. Furthermore, the identified KOGs transcripts of CaTA v2 (452) were also checked in above three legume transcriptomes to evaluate the completeness of annotation. It was observed that all 452 were mapped in common bean followed by 451 in soybean and 396 in *Medicago.*

### Mapping of CaTA v2 onto the *Medicago truncatula* Genome

All chickpea transcriptome assembly contigs (TACs) of the CaTA v2 were aligned to *Medicago truncatula* genome v3.5.1 (http://medtr.comparative-legumes.org/gb2/gbrowse/3.5.1/) [21] using the alignment program Exonorate 2.2.0 [27], requiring alignments of at least 80% identity and 50% coverage. TACs hitting more than 10 times to the *Medicago* genome were considered repeats, and discarded. Of 46,369 TACs, 20,119 (43.4%) could be aligned. All the alignments can be viewed in the Legume Information System (LIS) genome browser at http://medtr.comparative-legumes.org/gb2/gbrowse/Mt3.5.1.http://bit.ly/UHnDbD - _blank Chickpea transcripts matched to 12,484 *Medicago* genes, and are well distributed on all eight *Medicago* chromosomes, ranging from 18 to 31% of genes per chromosome (Table 3).

**Table 3:** Mapping of chickpea TACs onto *Medicago* genome.

*Medicago* (Mt) Chromosomes	Total CaTA v2 hits	Hit in non-genic region (where hit has not overlapped genicregion)	Hit in genic region	Genescovered	Total number of geneson each chromosomes	Percentage of gene covered on each chromosomes
Mt01	3,112	153	2,959	1,437	4,585	31.34
Mt02	3,435	95	3,340	1,488	5,022	29.63
Mt03	4,388	120	4,268	1,789	5,858	30.54
Mt04	4,380	113	4,267	2,083	6,529	31.90
Mt05	4,406	93	4,313	2,096	7,274	28.81
Mt06	1,861	54	1,807	537	2,840	18.91
Mt07	3,423	80	3,343	1,667	5,524	30.18
Mt08	2,888	99	2,789	1,387	4,486	30.92
**Total**				**12,484**	**42,118**	**29.64**

**Figure 4:**
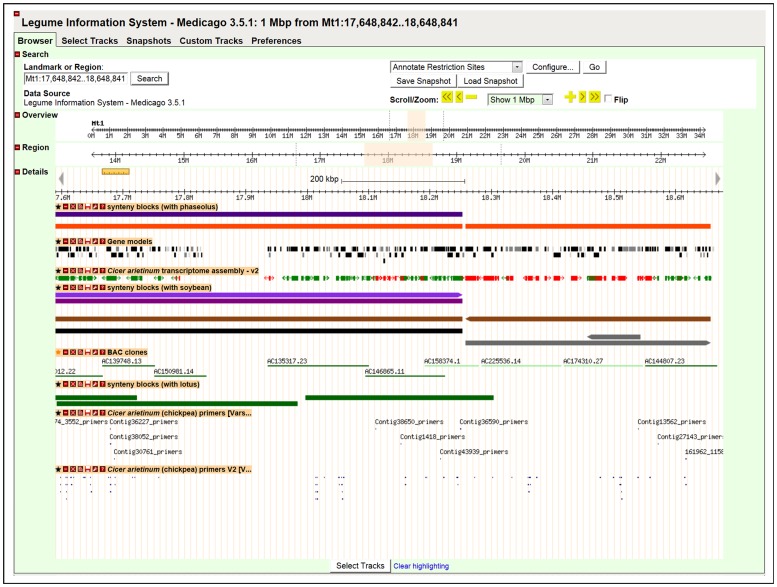
A sample view of chickpea TACs, markers and candidate ISR markers onto *Medicago* Genome sequence. This image is from Legume Information System (LIS) GBrowse viewer at http://medtr.comparative-legumes.org/gb2/gbrowse/3.5.1/, shows 1 Mb (17,648,842.18,648,841 of *Medicago*, chromosome Mt1). Red: There was at least one additional reported CaTA v2 alignment Green: There were no other reported alignments.

Out of 20,119 CaTA v2 TACs that were aligned with the *Medicago* genome, 15,263 (75.8%) and 2,919 (14.5%) had one and two matches, respectively. Details of the number of chickpea TACs mapped onto *Medicago* at a given number of times are given in Table 4. Synteny between chickpea and *Medicago* genes can be visualized genome-wide in the LIS genome browser at http://medtr.comparative-legumes.org/gb2/gbrowse/3.5.1/http://bit.ly/UHnDbD - _blank (Figure 4).

**Table 4:** Multiple mapping of CaTA v2 onto *Medicago* genome.

Number of times mappedto *Medicago* genome	Number of CaTA
1	15,263
2	2,919
3	772
4	370
5	218
6	356
7	83
8	67
9	46
10	25
**Total**	20,119

### Transcript Sequences Derived SSR and ISR Markers

All TACs (46,369) were mined for the presence of SSRs (di- to hexa- nucleotide motif) with the MIcroSAtellite (MISA) tool [28], giving 5,342 SSRs in 4,373 TACs (Table 5). The most frequently occurring di-nucleotide motifs were AG (478) followed by TC (456) and CT (295), whereas among tri-nucleotides GAA (214) is the highest followed by TTC (172). With an objective to convert the identified SSRs into potential genetic markers, an attempt was made to design the primer pairs for the TACs containing SSRs. Primer pairs could be designed for 2,474 SSRs corresponding to 2,231 TACs (Table S2).

**Table 5:** Identification of simple sequence repeats: their distribution and primer design for chickpea genetics and breeding applications.

Total number of sequences examined	46,369
Total size of examined sequences (bp)	44,740,166
Total number of identified SSRs	5,342
Number of SSR containing TACs	4,373
Number of TAC containing more than 1 SSR	734
Number of SSRs present in compound formation	472
*Distribution to different repeat type classes (excluding mono-nucleotide repeats)*	
Number of di-nucleotide repeats	2,094
Number of tri-nucleotide repeats	2,993
Number of tetra-nucleotide repeats	113
Number of penta-nucleotide repeats	56
Number of hexa-nucleotide repeats	86
*Primer pairs for SSRs*	
TACs were used to design primer pairs	2,231
Total numbers of primer pairs designed	2,474

The alignment of the CaTA v2 transcriptome assembly with the *Medicago* genome predicted 14,292 intron spanning regions (ISRs), for a total of 5,786 TACs. After removing hits >10 as discussed above under the alignment criteria, 14,153 ISRs for a total of 5,746 TACs could be identified. Of these, 2,473 TACs had only 1 ISR markers, while the remaining 3,273 TACs had >2 ISR markers. The alignments and primer sets can be viewed on the LIS genome browser at http://medtr.comparative-legumes.org/gb2/gbrowse/3.5.1/and are available for download at http://data.comparative-legumes.org/transcriptomes/cicar/lista_cicar-201201. A minimum of one and a maximum of 100 ISRs were designed against each matched *Medicago* gene (varying based on the number of introns in a gene and the ability of the primer prediction software to identify low-copy ISR markers across the introns). The longest contig contained five ISR markers while the shortest had three ISRs.

### Syntenic Relationship between Chickpea and *Medicago*

Putative mapping positions for a set of these markers were predicted based on syntenic regions between chickpea and *Medicago* genomes, using 555 genic molecular markers (10 CGMM - Chickpea Genic Molecular Markers, 12 ICCMs – ICRISAT Chickpea Markers, 15 CISR- Chickpea Intron Spanning Region markers, 262 CKaMs – Chickpea KASPar Assay Markers, 256 TOGs - Tentative Orthologous Gene markers) in the chickpea genetic map as anchor points [9], [11]. Of these 555 loci, 553 showed synteny in *Medicago* chromosomes. The strongest associations were for Mt_Chr3 and CaLG05 with 74 markers. Followed by Mt_Chr2 and Mt_Chr1 for CaLG 01 and CaLG 04; for these associations, there were 62 and 58 ISR markers in syntenic regions, respectively (Table 6). In this context, 553 chickpea GMMs mapped onto the genome sequence of *Medicago* were used to identify putative linkage groups for 14,153 chickpea ISRs. Putative linkage groups for 12,109 chickpea ISRs have been identified corresponding to 4,990 TACs. This method produced putative linkage group assignments for all eight of the chickpea linkage groups. Details of correspondences of ISR markers between *Medicago* chromosome and expected chickpea CaLGs are given in Table 7 and the distribution of ISR markers on chickpea TACs is given in Table 8.

**Table 6:** Correspondences of chickpea genic molecular markers to *Medicago*.

Chickpea linkage groups	Chickpea uniqueloci (no.)	Mt1	Mt2	Mt3	Mt4	Mt5	Mt6	Mt7	Mt8	Mtx	Total
CaLG01	69	0	62	1	2	0	0	0	0	2	67
CaLG02	61	1	0	1	2	43	8	4	0	2	61
CaLG03	62	2	0	0	3	2	1	50	1	3	62
CaLG04	95	58	0	5	11	8	3	4	0	6	95
CaLG05	93	3	0	74	2	4	2	2	1	5	93
CaLG06	76	0	2	3	35	4	2	2	24	4	76
CaLG07	46	1	0	1	35	1	0	0	8	0	46
CaLG08	53	0	0	0	2	42	4	4	0	1	53
**Grand Total**	**555**	**65**	**64**	**85**	**92**	**104**	**20**	**66**	**34**	**23**	**553**

**Table 7:** Distribution of ISRs on chickpea linkage groups.

Chickpea linkage group	Number of ISR markers showing inferred position	Markers selected for analysis	Markers amplified
CaLG01	1,773	21	8
CaLG02	1,257	20	14
CaLG03	1,216	20	5
CaLG04	1,643	23	11
CaLG05	1,764	25	5
CaLG06	2,203	13	7
CaLG07	1,392	20	4
CaLG08	861	16	2
**Total**	**12,109**	**158**	**56**

**Table 8:** Distribution ISRs on chickpea contigs.

Number of contigs	Number of ISR	Total ISRs
2,473	1	2,473
1,342	2	2,684
771	3	2,313
468	4	1,872
265	5	1,325
154	6	924
88	7	616
70	8	560
32	9	288
34	10	340
14	11	154
9	12	108
3	13	39
10	14	140
4	15	60
2	16	32
3	19	57
1	20	20
1	21	21
1	27	27
1	100	100
**5,746**		**14,153**

### Validation and Polymorphism of Novel ISR Markers

Primer pairs were designed for 12,109 ISRs; however, a subset of 4,990 ISR markers, each corresponding one intron spanning region to one TAC, were selected for further analysis. From this subset of 4,990 primer pairs, 158 ISR markers were selected for validation purpose. These 158 ISR markers were selected from all eight CaLGs to represent genome-wide distribution.

All 158 primer pairs were screened for amplification of DNA from five chickpea genotypes, namely ICC 4958, ICC 1882, ICC 283, ICC 8261 and PI 489777. The five genotypes correspond to the parents of different mapping populations. This analysis identified a set of 56 markers (35%) with scorable amplicons in all five genotypes. These 56 ISR markers corresponded to all eight chickpea linkage groups with maximum number of markers on CaLG02 (14) and minimum number of markers on CaLG08 (2). Screening of these amplified 56 ISRs on five chickpea genotypes, including four cultivated (ICC 4958, ICC 1882, ICC 283 and ICC 8261) and one wild (PI 489777) line, showed length polymorphism (two to three alleles) with 11 (20%) markers (Table S3). The polymorphism information content (PIC) value for the polymorphic markers ranged from 0.26 to 0.36, with an average of 0.27.

## Discussion

Recent years have witnessed significant advances in the development of genomic resources to support molecular breeding in chickpea. As a result, a range of sequencing platforms were used and different sets of transcriptome assemblies were developed [10], [12], [15], [16], [25]. This study aimed to generate a comprehensive transcriptome assembly based on a hybrid approach in order to precisely characterize the chickpea transcriptome. A hybrid assembly comprising of 46,369 using transcriptome sequence acquired from three data sets composed of NGS (FLX/454 and llumina) and Sanger sequencing ([10], [12], [15], [16], [20], NCBI) data was generated.

Earlier assemblies were developed based on CAP3 [10], [15], [16] and TGICL [12] programs, while the present hybrid assembly has been developed using three effective assembly programs, Newbler, ABySS and MIRA that can accommodate large amounts of short sequences generated by next-generation sequence technologies [22], [23] (Table 2). Therefore the developed assembly is a hybrid/comprehensive assembly. Drawbacks from single sequencing platforms may be compensated for by different characteristics of sequences from other platforms. Hence hybrid assemblies using a combination of datasets are demonstrated superior to assemblies generated using sequence data generated from one sequencing platform [12], [29], [30]. Furthermore, the hybrid assembly developed in this study comes from >22 tissues representing a range of developmental stages and 8 tissues challenged by different stresses from >17 genotypes. The CEGMA pipeline [24] was used to assess the completeness of CaTA v2; the presence of >98% KOGs in CaTA v2 showed that the developed transcriptome assembly has captured almost the complete gene space in chickpea. CEGMA pipeline was used to check the completeness of gene space in several genome sequencing projects e.g. chickpea [18], pigeonpea [31] as well as transcriptome assembly of several plant species such as *Nicotiana benthamiana* [32] etc.

The completeness and quality of this assembly was assessed by comparing it with itself and also with earlier transcriptome assemblies (Table 2) [10], [12], [16], [20], [25]. For instance, when the datasets were analyzed individually in earlier studies, a wide range of TAC or TUS counts were reported: 43,389 contigs from 1.8 million FLX/454 reads and 121 million Illumina reads [25]; 74,651 contigs from 107 million Illumina reads [20]; 34,760 contigs from 2 million FLX/454 reads and 107 million Illumina reads [12]; 638 contigs from 5,494 Sanger reads [16] and 1,590 contigs from 20,162 Sanger ESTs [15]. The CaTA v1 [10], assembled from 435,018 FLX/454 reads and 21,491 Sanger ESTs, produced an assembly of 103,215 TUSs, of which 44,845 were contigs and 58,370 were singletons. The CaTA v2 has a total of 46,369 TACs, with N50 of 1,726 bp, while the CaTA v1 included 44,845 contigs, with N50 length of only 515 bp [10]. In terms of assessment of redundancy, the CaTA v2 assembly was found to contain 137 redundant contigs at 100% similarity analysis. However, this is not unexpected as some redundant sequences are likely to be present in the hybrid assembly, when sequence data generated on different platforms are used for assembly. Such redundancy has been observed in other studies. For example at 100% similarity, the ICCV 2 transcritome assembly by Agarwal et al. [25] has higher (457) redundant contigs. Other assemblies from Garg et al. [12] and Hiremath et al. [10] also have 287 and 105 redundant contigs, respectively. As compared to all these assemblies, the CaTA v2 assembly has less redundancy. Therefore the hybrid transcriptome assembly (CaTA v2) has several improved features than earlier versions. Thus, this assembly (CaTA v2) can be considered the most comprehensive transcriptome assembly of chickpea.

Sequence annotation of the chickpea TACs of CaTA v2 based on BLASTX using the nonredundant UniProt database showed significant functional annotation for 70.8% of the TACs. This high percentage of similarity favors understanding of the biology and identification of candidate genes in under-studied crops like chickpea. In addition, this information may facilitate genomic analyses like gene expression and can provide the information about gene content and function, particularly gene discovery and identification of candidate genes, for development of molecular markers [10], [15], [16]. Furthermore, TACs identified under specific categories like response to stimulus and enzyme classification could serve as a useful source to identify stress responsive genes and genes involved in different metabolic pathways for chickpea crop improvement. In addition, a total of 7,722 putative chickpea transcription factor genes, distributed in 83 families, identified in the new assembly represent 16.65% of transcripts. Whereas earlier assembly by Garg et al. [20] identified 57 transcription families representing 12.3% transcripts. The predicted transcription factor encoding genes in other legumes like *Medicago* (1,473), *Lotus* (1,637) [33] and soybean (5,671) [34] are less than the TF genes identified in the present study. Hence future studies on the TF gene families identified in the present study may contribute to disclose gene regulatory mechanisms in chickpea and related legumes.

Mapping of CaTA v2 with *kabuli* and *desi* genomes revealed that the *kabuli* genome is more comprehensive (98% TACs mapped) when compared to the *desi* genome (90% of TACs mapped). These results were supported by coverage of the annotated gene set of *kabuli* and *desi* genomes by CaTA v2. Coverage of the *kabuli* gene set (91%) was much higher than the *desi* gene set (69%). Coverage of *desi* genome still remains low even when compared with the unigene set generated from all five transcriptome assemblies. The reason behind these uncovered genes could be due to three main possibilities - i) low expressed genes could not be captured at the time of cDNA synthesis, ii) incorrect annotation of genes (eg. pseudogenes) in the *desi* genome, and iii) chromosomal segments with uncovered genes may not be from chickpea. Further investigation of *desi* and *kabuli* genomes by using sequence reads from isolated chromosomes indicated that some regions of the *desi* pseudomolecules do not reflect the physical content of the *desi* genome (Ruperao et al., unpublished data).

Additionally, conservation across legume species has been revealed by comparative analysis with other legumes like *Medicago,* soybean and common bean and the developed CaTA v2. High degree of conservation across legume has been reported earlier [18], [20]. Maximum TACs were aligned to soybean followed by common bean and *Medicago* which was not expected in the evolutionary relationship point of view [18]. This could be due to incomplete genome sequence of *Medicago* or incomplete annotation of the genome. This was further supported by low number of KOGs in *Medicago* when verified by the alignment of 452 KOGs identified in CaTA v2 to other legumes.

ESTs have been utilized for large-scale gene discovery and marker development in many plants and crop species. This study resulted in several large new SSRs and ISR marker sets for chickpea. As these markers are derived directly from coding parts of the genome, they provide good opportunities to identify the ‘perfect marker’ for traits of interest for enhancing the precision of efficiency of molecular breeding in chickpea. EST/ transcript-derived SSRs have been widely used in constructing high-density linkage maps, marker-trait association, diversity analysis, etc. in several crop species [35]. As transcripts are more highly conserved than nongenic sequence, they are useful in detecting the signature of divergent selection [36].

The closest sequenced species to chickpea is *Medicago* which diverged ∼10–20 million year ago [37]. One major application of the transcriptome assembly in development of genome-wide marker datasets for enriching the genetic map of chickpea, using a comparative genomics approach that employs the *Medicago* genome sequence [21] and the genic molecular marker loci based genetic map of chickpea, has been demonstrated by Hiremath et al. [9]. Comparison of the CaTA v2 with *Medicago* genome identified the homologues for 60% of the chickpea TACs, and covering 12,484 genes in the *Medicago* genome. Of these, the majority of TACs (15,263/20,119) mapped once against *Medicago* genome.

Furthermore, alignment of CaTA v2 TACs against *Medicago* helped in identification of ISR markers within the limit of exon-intron regions. 14,153 ISRs derived from the 5,746 TACs can be studied for length polymorphism between parents of different mapping populations. To identify effective makers for molecular breeding in chickpea, a set of markers were short-listed based on synteny results of 553 anchor points corresponding to genic molecular markers in chickpea established through sequence homology with the *Medicago* genome. Based on this information, 12,109 ISR markers were identified that have putative chromosomal placements in the chickpea genome. A subset (158) of these markers was further analyzed for length (*indel*) polymorphism in 5 parental genotypes of mapping populations segregating for important biotic (e.g *Helicoverpa*) and abiotic (e.g. drought) stresses. While 56 markers provided scorable amplicons, 11 markers showed polymorphism with 2–3 alleles in the genotypes analyzed on MDE gel [38]. As expected, seven out of 11 markers showed polymorphism with PI 489777, a wild species, while only 4 markers showed polymorphism within cultivated genotypes. Low levels of polymorphism between cultivated species has been reported in other crops [39]–[42]. Validation results as well as polymorphism information with ISR markers on MDE gel emphasize the importance of ISR markers. These markers should be a good resource for genetic mapping and trait mapping in chickpea breeding programs.

In conclusion, the present study demonstrated a high-quality comprehensive transcriptome assembly representing *kabuli* and *desi* varieties of the important legume crop chickpea using Sanger and second-generation sequencing (FLX/454 and Illumina) technologies. Developed transcriptome assembly CaTA v2 and marker resources will not only help chickpea breeding programs to identify elite varieties leading to increased crop productivity, but also will convey novel information for future genetic studies in chickpea. Functional annotation and identification of syntenic regions between the chickpea and a related legume, *Medicago*, provide greater insight into the chickpea gene content. The identified ISR and SSR markers will help improve marker density, and as a result these markers will be useful in chickpea breeding programs. The next step is deployment of the developed genomic resources described in this study in breeding programs for genetic enhancement and development of elite breeding lines.

## Materials and Methods

### Sequence Datasets

The following three datasets were used for defining the transcriptome assembly: (a) 134.95 million Illumina short single-end reads generated from the ICC 4958 genotype at NIPGR [20], referred as Dataset I; (b) 7.12 million FLX/454 reads generated from nine genotypes at ICRISAT/J. Craig Venter Institute (JCVI) and The National Research Council Canada (NRC-CNRC) [10], [12], referred as Dataset II; and (c) 139,214 vector-trimmed Sanger ESTs downloaded from dbEST (http://www.ncbi.nlm.nih.gov/dbEST/) (the majority of which were generated at ICRISAT, [15], [16]) referred as Dataset III (see Table 1).

### Sequence Assembly

Sequence datasets, as mentioned above, were assembled using the programs ABySS [22], Newbler (http://www.454.com/products/analysis-software/) and MIRA [23], using the following three steps. In the first step, all Illumina reads were assembled together using ABySS. In the second step, FLX/454 reads from nine genotypes (Datasets II) were trimmed of adapter sequences and assembled individually using the Newbler assembler. Subsequently, the pooled Illumina (step 1 by ABySS) and FLX/454 (step 2 by Newbler) assemblies were merged with vector-trimmed Sanger ESTs of Dataset III using the MIRA program. All programs were run with the default settings, except for the following parameters: for ABySS, scaffolding ‘on’ at the paired-end stage; and for MIRA these options specified as “no”: Load straindata, Enforce presence of qualities, Extra gap penalty and Wants quality file. In order to decrease runtime, number of processors used was 7. Since we were interested in a consensus assembly, the “Load straindata” option was turned off. During the second stage of the assembly where FLX/454 and Sanger ESTs were merged, there were no quality scores for the Illumina contigs. Therefore “Enforce presence of qualities” and “Wants quality file” options were specified to “no”. By turning off “Extra gap penalty”, we avoided penalizing gaps during the Smith-Waterman alignment, especially since FLX/454 data is known to have homopolymer errors. Microbial contamination and rRNA contamination tags were searched against NCBI bacterial genomes database (ftp://ftp.ncbi.nlm.nih.gov/genomes/Bacteria/) as well as rRNA collected from other crops from NCBI’s database. For checking the completeness of the transcriptome assembly, the core eukaryotic gene-mapping approach (CEGMA) pipeline [24] was used.

### Comparison of CaTA v2 to Itself and Other Chickpea Transcriptomes

BLASTN from NCBI BLAST+ (ftp://ftp.ncbi.nlm.nih.gov/blast/executables/blast/) was used to compare CaTA v2 to itself. Alignment with different query and subject were filtered at 95% identity and 99% coverage and were regarded as redundant. CD HIT package (http://www.bioinformatics.org/project/filelist.php?group_id=350; Version 4.5.4) was used for clustering of transcripts from reported chickpea transcriptome assemblies [10], [12], [20], [25] as well as the developed CaTA v2. Transcripts were clustered using cd-hit-est program with a sequence identity cut-off of 0.9.

### Functional Annotation and Similarity Search

Functional annotations of 46,369 TACs were made using BLASTX comparisons against the UniRef90 (ftp://ftp.uniprot.org/pub/databases/uniprot/uniref/uniref90/), a non-redundant protein data set from the UniProt database. Each chickpea transcript was tentatively assigned the function of the best hit (E-value 1e-06) using UniProt Knowledgebase (UniProt KB; http://www.uniprot.org/). Subsequently, TACs that showed a significant BLASTX hit were used for functional annotation based on Gene Ontology (GO) categories from the UniProt database (UniProt-GO; http://www.uniprot.org/). TACs were thus assigned to primary and sub-GO functional categories. To identify the transcription factors in CaTA v2, we compared the assembly to plant-specific transcription factor database PlnTFDB (http://plntfdb.bio.uni-potsdam.de) using BLASTX search with stringency of E-value 1e-06.

### Comparison of CaTA v2 to *kabuli* and *desi* Reference Genomes and Other Legume Transcriptomes

CaTA v2 TACs were aligned on to chickpea reference genome assemblies, *kabuli* [18] and *desi* [19] using BLAT program [43]. Best alignments were selected using script “pslSort”.While gene sets of both genome assemblies with CaTA v2 and unigene set from five transcriptome assemblies were compared using BLASTN of standalone package of NCBI BLAST+ (ftp://ftp.ncbi.nlm.nih.gov/blast/executables/blast/) at a lower stringency with E-value, 1e-06. For mapping Illumina short sequence reads onto *desi* gene set, sequence library of the *desi* genotype ICC 4958 (SRR063784) containing 31.02 million reads was downloaded from NCBI SRA database. Filtered high quality reads (after discarding low quality reads) were mapped using SOAP2 [44] through Integrated SNP mining and Utilization (ISMU) pipeline (Azam et al., unpublished).

Annotated transcriptomes of three legumes, *Medicago*, soybean and common bean were downloaded from Phytozome database (www.phytozome.net) and compared with CaTA v2 using BLASTN from NCBI BLAST+.

### Identification of Microsatellite/SSRs

SSR mining of 46,369 TACs was carried out using the MIcroSAtellite (MISA) search tool [28]. Parameters used were: at least 6 repeats for dinucleotide and 5 repeats for tri-, tetra-, penta- and hexanucleotide for simple SSRs. Both perfect (i.e. SSRs containing a single repeat motif such as ‘AGG’) and compound (i.e. composed of two or more SSRs separated by < = 100 bp) SSRs were identified. The Primer3 program [45] was used for designing the primer pairs based on the following criteria: annealing temperature (Tm) between 50–65°C with 60°C as optimum, product size ranging from 100 bp to 350 bp, primer length ranging from 18 bp to 24 bp with an optimum of 20 bp and GC % content in the range of 40–60%.

### Mapping of the Chickpea Transcriptome Assembly onto *Medicago* Genome

All TACs of CaTA v2 assembly were aligned to *Medicago* genome v3.5.1 (http://medtr.comparative-legumes.org/gb2/gbrowse/3.5.1/) using Exonerate 2.2.0 [27], with parameters and flags “percent 25” (to report only alignments over 25% of the maximum score attainable by each query) and “refine region” (to perform an exhaustive alignment over the region in which the heuristic alignment was found). Alignments were filtered to require at least 80% alignment identity and 50% query coverage. If this resulted in more than 12 matches for a given sequence, the sequence was considered repetitive, and all matches were discarded.

### Mapping of Genic Molecular Marker Loci of Chickpea onto the *Medicago* Genetic Map

Genic molecular marker loci genetically mapped in chickpea [9] were anchored to the *Medicago* genome using BLASTN [46] with maximum E-value 1e-08, followed by manual selection for best hits matching up to two homoeologous *Medicago* regions.

### Identification of Intron Spanning Region (ISR) Markers

Alignment results of chickpea TACs with the *Medicago* genome were analyzed for identification of flanking intron junctions. The Exonerate alignment of the TACs, in Exonerate “vulgar” (Verbose Useful Labeled Gapped Alignment Report) output format, was used to identify intron junctions in the TAC sequences. These junctions were used to design the primer pairs using Primer3 [45] and BatchPrimer3 [47]. Primer pairs were re-mapped to the *Medicago* genome (to evaluate for repetitive sequences) using e-PCR [48], with parameters “-n3 -g1 -t3 -m400 -d50-1000”. These parameters have the following effects: “-n3” allows up to three mismatches per primer; “-g1” allows up to one gap per primer; “-t3” specifies output in tabular format; “-m400” specifies an allowable margin for the product of 400 bases; and “-d50-1000” specifies the default PCR product size range. Primer pairs with more than two alignments at these parameters were discarded.

Putative approximate mapping positions for the identified ISR markers were imputed based on anchoring points between chickpea and *Medicago* genetic maps using genic molecular marker loci of chickpea. Where there are two or more chickpea marker loci with proximity in both chickpea and *Medicago* (i.e. with nearby cM values in chickpea and nearby nucleotide positions in *Medicago* chromosome pseudomolecules), tentative chickpea linkage groups (CaLGs) were assigned for ISR candidate markers occurring between the neighboring chickpea genic molecular markers.

### ISR Analysis

Polymerase chain reactions (PCRs) for amplification of ISR loci were performed on five chickpea genotypes (4 cultivated and 1 wild species) in a 5 µl reaction volume as described by Gujaria et al. [17]. Amplified products were denatured and separation was undertaken on MDE gel electrophoresis as described earlier [38]. Polymorphic information content (PIC) value has been obtained using PowerMarker v3.25 [49].

## Supporting Information

Table S1 **Assessment of transcript coverage on** ***kabuli*** **and** ***desi*** **genome assemblies.**
(XLSX)

Table S2 **Details of designed primer pairs identified from chickpea transcriptome assembly- CaTA v2.**
(XLS)

Table S3 **Allele scoring of ISR markers amplicons between the selected chickpea genotypes.**
(XLS)
